# Real-World Data of Combined Immunochemotherapy in Patients With Nonsquamous Advanced NSCLC. A Single-Center Retrospective Study

**DOI:** 10.1016/j.jtocrr.2023.100509

**Published:** 2023-03-24

**Authors:** Till Wallrabenstein, Maximilian Mamot, Eveline Daetwyler, David König, Sacha I. Rothschild

**Affiliations:** a Department of Medical Oncology and Comprehensive Cancer Center, University Hospital Basel, Basel, Switzerland; b Department of Hematology and Oncology, University Medical Center Freiburg, Freiburg, Germany; c Center of Oncology/Hematology and Comprehensive Cancer Center, Cantonal Hospital Baden, Baden, Switzerland

**Keywords:** Non–small cell lung cancer, Immuno-chemotherapy real-world data, Pembrolizumab, ECOG PS 2

## Abstract

**Introduction:**

On the basis of the landmark trial KEYNOTE-189 (KN-189), pembrolizumab plus chemotherapy has become the standard-of-care first-line treatment for patients with advanced nonsquamous NSCLC without oncogenic driver alterations.

KN-189 included a selected patient population and lacks external validity. In clinical practice, many patients do not meet the inclusion criteria of KN-189, although they are treated accordingly. It is unknown whether these patients benefit equally as the trial population.

**Methods:**

We retrospectively analyzed all patients with advanced nonsquamous NSCLC without targetable oncogenic alterations who received the KN-189 treatment regimen between April 2018 and May 2021 at the University Hospital Basel, Switzerland. Patients were grouped into those who retrospectively met the inclusion criteria of KN-189 (group A) and those who did not (group B). Outcome parameters included progression-free survival (PFS), overall survival (OS), and objective response rate. Multivariate subgroup analyses were performed.

**Results:**

We identified 75 patients, including 29 patients in group A and 46 patients in group B. Median PFS was 9.2 and 4.6 months in group A and B, respectively (*p* = 0.12). Median OS was 16.5 and 6.5 months in group A and B, respectively (*p* = 0.11). Objective response rate was 59% in group A and 33% in group B (*p* = 0.03). Eastern Cooperative Oncology Group performance status greater than or equal to 2 and active infections were significantly associated with shorter PFS and OS.

**Conclusions:**

We report real-world data for patients treated according to the KN-189 regimen with inferior outcomes in patients who did not meet the KN-189 inclusion criteria. Better treatment options for this vulnerable patient population are needed.

## Introduction

Lung cancer is a major global health care burden. It is the most frequently diagnosed cancer and the most frequent cancer-related cause of death with an estimation of more than 228,000 new cases and more than 135,000 attributable deaths in 2020 in the United States alone.^1^ More than half of all patients are diagnosed with having stage IV disease.^2^

The treatment landscape for advanced/metastatic NSCLC has evolved dramatically in the past two decades. Although platinum-based doublet chemotherapy was the standard of care for many years, the identification of targetable oncogenic driver alterations and the introduction of immune checkpoint inhibitors have considerably changed the therapeutic landscape of NSCLC.^3^, ^4^, ^5^, ^6^

For patients with advanced nonsquamous NSCLC without targetable mutations, the pivotal phase 3 trial KEYNOTE-189 (KN-189) has defined a new treatment standard. Compared with four cycles of cisplatin/carboplatin plus pemetrexed maintenance, the addition of pembrolizumab was found to increase progression-free survival (PFS) from 4.9 to 8.8 months and overall survival (OS) from 10.6 to 22 months.^7^^,^^8^ The results of KN-189 are outstanding even in comparison to other randomized trials investigating the addition of immunotherapy to chemotherapy in advanced NSCLC, raising the question of a highly selected patient population.

KN-189 and other pivotal phase 3 trials have highly selective inclusion criteria. Our observation is that many of our patients seen in daily clinical practice are not fulfilling these criteria, especially patients with lung cancer who often present with a poor performance status and relevant comorbidities. Because they are underrepresented in clinical trials, we lack strong evidence on how to treat vulnerable patient groups, such as elderly and comorbid patients or patients with a poor performance status. There is increasing evidence that also older patients, patients with a poor performance status, and patients with relevant comorbidities might benefit from immune checkpoint inhibitors.^9^, ^10^, ^11^ Nevertheless, the optimal treatment regimen for these patients is unknown and there is lack of clinical evidence from prospective randomized clinical trials. Retrospective real-world data about these patient populations are also limited. Trying to meet their needs, often we decide to treat these patients according to certain protocols nevertheless, assuming they may benefit in an equal manner as trial populations.

Here, we report a retrospective analysis of all patients with advanced nonsquamous NSCLC who received treatment according to the KN-189 regimen at the University Hospital Basel, Switzerland, with retrospective consideration of trial eligibility.

## Materials and Methods

### Study Design and Patient Population

We retrospectively collected and analyzed data from all patients with Union for International Cancer Control stage IV nonsquamous NSCLC without EGFR mutations or ALK/ROS 1/RET fusions who were treated using the KN-189 regimen between April 24, 2018, and May 30, 2021, at the University Hospital Basel, Switzerland. Patients were identified through a keyword search of the hospital's electronic medical record system and the chemotherapy prescription software.

Patients were divided into the following two groups: group A, patients who retrospectively met the inclusion criteria of KN-189 as defined by the trial protocol,^7^ and group B, patients who did not. Deviating from the eligibility criteria defined in the KN-189 protocol, we have established minor tolerance criteria regarding temporary exclusion factors that might have been reversed in a prospective setting. These criteria were radiotherapy to the brain or corticosteroid treatment for symptomatic brain metastasis continued during the first cycle of treatment and isolated anemia of nonstructural cause, reversed within 7 days after treatment initiation.

To provide a comprehensive real-world treatment experience, we have included patients having received previous systemic treatment for stage IV NSCLC (e.g., single-agent pembrolizumab) before starting the KN-189 regimen. Furthermore, patients who have not received at least one dose of pembrolizumab before reaching one of the end points were included (retrospective intention-to-treat). We have provided an additional modified analysis that excludes pretreated patients and patients not having received at least one dose of all three drugs (platinum, pemetrexed, pembrolizumab).

This study was approved by the local ethics committee (ethics committee of northwestern and central part of Switzerland, EKNZ) (project ID 2021-01487). In accordance with the Human Research Act on the basis of article 118b paragraph 1 of the Federal Constitution, all patients included in this analysis signed a general consent that regulates the use of clinical data for research purposes.

### End Points

Analyzed patient outcome parameters were PFS, OS, and objective response rate (ORR). PFS was defined as time from treatment initiation to radiographic progression documented during routine scans according to Response Evaluation Criteria in Solid Tumors (RECIST) version 1.1, death, or loss to follow-up.^12^ OS was defined as time from treatment initiation to death or loss to follow-up. Data cutoff was on October 14, 2021. ORR was defined according to RECIST version 1.1 and was evaluated during routine scans.

Planned subgroup analyses were performed for age, sex, histological subtype, Eastern Cooperative Oncology Group performance status (ECOG PS), syn- versus asynchronous metastatic disease, brain metastases, prior systemic treatment, prior surgery, prior radiotherapy, use of corticosteroids, and use of antibiotics.

### Statistical Analysis

Baseline and treatment characteristics were compared by Fisher’s exact test for discrete variables and Mann-Whitney *U* test for continuous variables. Time-to-event end points were calculated by Kaplan-Meier method and compared using log-rank test. Median follow-up time was calculated by using the reverse Kaplan-Meier method. Multivariate subgroup analyses were performed by Cox regression on time-to-event end points and by binary logistic regression on discrete end points (ORR). A *p* value less than 0.05 was considered significant. No correction for multiple testing was applied. All statistical analyses were performed using SPSS version 28 (IBM Corp., Chicago, IL).

## Results

### Patient and Disease Characteristics

Overall, 75 patients with advanced nonsquamous NSCLC without EGFR mutation or ALK/ROS1 fusion have started treatment according to the KN-189 regimen between April 2018 and May 2021 at the University Hospital Basel.

**Figure 1:**
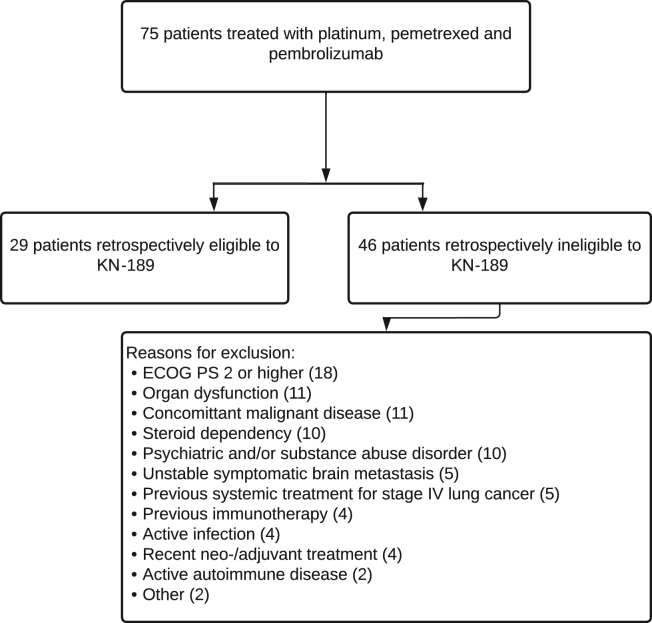
Group division. A total of 75 patients with metastatic NSCLC treated with platinum, pemetrexed, and pembrolizumab were identified by keyword search and retrospectively divided into two groups. There were 29 patients (group A) who would retrospectively have fulfilled the selection criteria of the KN-189 trial. There were 46 patients (group B) who would not have been eligible for KN-189 for reasons provided. ECOG PS, Eastern Cooperative Oncology Group performance status; KN-189, KEYNOTE-189.

Of all patients, 29 patients met the eligibility criteria of the KN-189 trial (group A). The remaining 46 patients (group B) met at minimum one definite trial exclusion criterion, including 33 patients with multiple exclusion criteria (Fig. 1). Main reasons for not meeting the KN-189 trial inclusion criteria were ECOG PS greater than or equal to 2, organ dysfunction as per-study protocol, concomitant malignant disease, use of systemic corticosteroids, and psychiatric or substance abuse disorder.

Baseline and disease characteristics of groups A and B are provided in Table 1. Median age and smoking status were equally distributed between groups. As a trend, there were more male patients and patients with large cell/not otherwise specified histology in group B.

**Table 1:** Baseline and Ailment Characteristics

Characteristics	Group A (n = 29)	Group B (n = 46)	Total (N = 75)	*p*
Median age, n (%)	68.7	67.1	67.4	0.56
Female	15 (51.7)	14 (30.4)	29 (38.7)	
Male	14 (48.3)	32 (69.6)	46 (61.3)	0.09
Histology, n (%)				
Adeno	28 (96.6)	38 (82.6)	66 (88.0)	
Large cell/NOS	1 (3.4)	8 (17.4)	9 (12.0)	0.14
ECOG, n (%)				
0	19 (65.5)	11 (23.9)	30 (40.0)	<0.001
1	10 (34.5)	17 (37.0)	27 (36.0)	1
≥2	0 (0.0)	18 (39.1)	18 (24.0)	<0.001
Smoking status, n (%)				
Current or former	27 (93.1)	42 (91.3)	69 (92.0)	1
Never	2 (6.9)	4 (8.7)	6 (8.0)	
Relevant comorbidity, n (%)				
Cardiovascular	16 (55.2)	28 (60.9)	44 (58.7)	0.64
Pulmonary	8 (27.6)	17 (37.0)	25 (33.3)	0.46
Metastases, n (%)				
Bone	12 (41.4)	18 (39.1)	30 (40)	1
CNS	13 (44.8)	15 (32.6)	28 (37.3)	0.33
Lung	12 (41.4)	16 (34.8)	28 (37.3)	0.63
Adrenal gland	6 (20.7)	9 (19.6)	15 (20.0)	1
Pleural	5 (17.2)	10 (21.7)	15 (20.0)	0.77
Liver	5 (17.2)	7 (15.2)	12 (16.0)	1
Peritoneal/abdominal	2 (6.9)	6 (13.0)	8 (10.7)	0.47
Other sites	9 (31.0)	16 (34.8)	25 (33.3)	0.81
Number of metastatic sites, n (%)				
1–2	17 (58.6)	33 (71.7)	50 (66.7)	
≥3	12 (41.4)	13 (28.3)	25 (33.3)	0.32
PD-L1—tumor cells, n (%)				
<1%	14 (48.3)	27 (58.7)	41 (54.7)	0.48
1%–49%	12 (41.4)	11 (23.9)	23 (30.7)	0.13
≥50%	3 (10.3)	8 (17.4)	11 (14.7)	0.51
Initial UICC stage, n (%)				
I/II	1 (3.4)	4 (8.7)	5 (6.7)	0.64
III	3 (10.3)	4 (8.7)	7 (9.3)	1
IVA	7 (24.1)	13 (28.3)	20 (26.7)	0.79
IVB	18 (62.1)	25 (54.3)	43 (57.3)	0.63
Previous treatment in curative intent, n (%)				
Radiochemotherapy	1 (3.4)	1 (2.2)	2 (2.7)	1
Surgery	5 (17.2)	9 (19.6)	14 (18.7)	1
Neo-/adjuvant systemic therapy	3 (10.3)	6 (13.0)	9 (12.0)	1
Previous local treatment, n (%)				
Radiotherapy of the brain	12 (41.4)	15 (32.6)	27 (36.0)	0.49
Pleurodesis for symptomatic effusion	4 (13.8)	8 (17.4)	12 (16.0)	0.76
Metastasectomy before treatment initiation	5 (17.2)	7 (15.2)	12 (16.0)	1

*Note:* Baseline and disease characteristics of 75 patients with metastatic NSCLC treated with platinum, pemetrexed, and pembrolizumab. Patients in group A (n = 29) would retrospectively have fulfilled selection criteria of the KEYNOTE-189 trial. Patients in group B (n = 46) would have been trial ineligible.

CNS, central nervous system; ECOG, Eastern Cooperative Oncology Group; NOS, not otherwise specified; PD-L1, programmed death-ligand 1; UICC, Union for International Cancer Control.

### Outcome Parameters

After a median follow-up time of 16 months (95% confidence interval [CI]: 12.4–18), 56 of 75 patients had a PFS event and 44 patients have died. Median PFS in the overall population was 7 months (95% CI: 4.6–9.4), and median OS was 12.6 months (95% CI: 4.2–21.1). ORR in the overall population was 43%.

**Figure 2:**
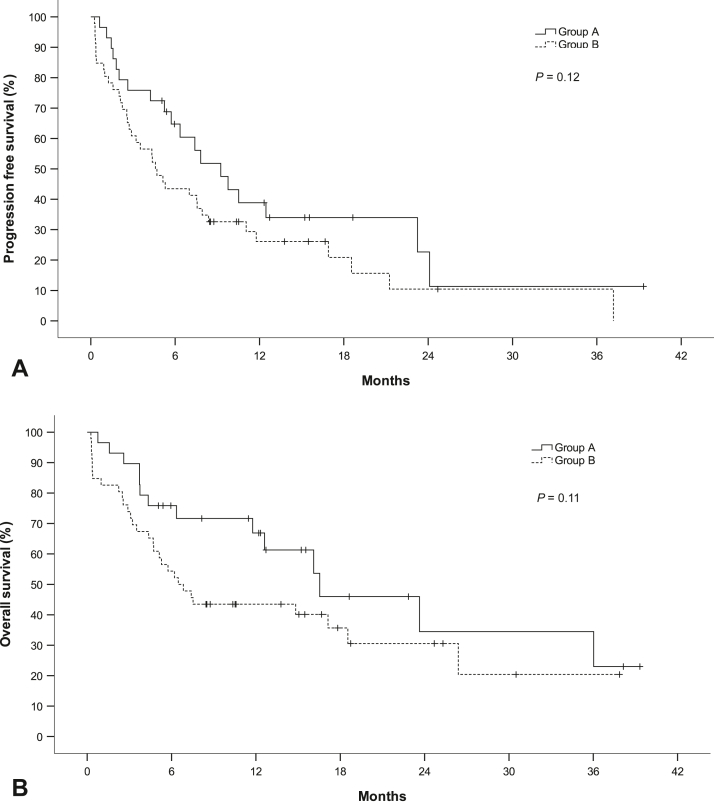
(*A*) Progression-free survival and (*B*) overall survival of 75 patients with metastatic NSCLC treated with platinum, pemetrexed, and pembrolizumab. Patients in group A (n = 29) would retrospectively have fulfilled selection criteria of the KEYNOTE-189 trial. Patients in group B (n = 46) would have been trial ineligible.

Median PFS was 9.2 months (95% CI: 5.7–12.8) in group A as compared with 4.6 months (95% CI: 2.6–6.6) in group B (*p* = 0.12; Fig. 2*A*). Median OS was 16.5 months (95% CI: 6.9–26.2) as compared with 6.5 months (95% CI: 4.0–9.0) in groups A and B, respectively (*p* = 0.11; Fig. 2*B*). Patients in group A had a significantly higher response (ORR = 59%) compared with patients in group B (ORR = 33%) (*p* = 0.03).

A total of nine patients died within 1 month after treatment initiation, including one patient in group A and eight patients in group B (*p* = 0.14). Early death was attributed to the treatment in two patients (one patient died owing to chemotherapy-induced febrile neutropenia, one patient had grade 5 immune-related colitis). The other patients died owing to tumor-related causes. Patients with early death tended to have a poor ECOG PS (ECOG PS ≥3 in four patients) at the time of treatment initiation.

There was no significant difference between groups regarding immune-related adverse events, chemotherapy-related toxicity, or toxicity-related treatment discontinuation. Patients in group B have received less cycles of platinum-based chemotherapy (average of 3 versus 3.4 cycles, *p* = 0.13), less cycles of pemetrexed maintenance therapy (average of 2.8 versus 4.3 cycles, *p* = 0.22), and less cycles of pembrolizumab (average of 7.3 versus 8.8 cycles, *p* = 0.16) than patients in group A (Supplementary Table 1).

### Subgroup Analysis

Patients with ECOG PS greater than or equal to 2 had a significantly shorter PFS (hazard ratio [HR] = 2.26, 95% CI: 1.04–4.9, *p* = 0.04) (Supplementary Table 2) and a trend toward inferior OS (HR = 2.3, 95% CI: 0.99–5.5, *p* = 0.054) (Supplementary Table 3) and ORR (OR = 0.26, 95% CI: 0.06–1.23, *p* = 0.09) (Supplementary Table 4) compared with patients with ECOG PS of 0 to 1.

Active infection at the time of treatment initiation was significantly associated with worse outcome in terms of PFS (HR = 20.7, 95% CI: 2.5–168.2, *p* = 0.005) (Supplementary Table 2) and OS (HR = 20.5, 95% CI: 2.1–197.8, *p* = 0.01) (Supplementary Table 3). This subgroup of patients includes four patients with pneumonia treated with intravenous antibiotics. Cause of death was respiratory failure in all cases. Only one of these four patients survived the first 2 weeks after initiation of chemoimmunotherapy and was treated with a second cycle. This patient experienced a recurrence of pulmonary infection, developed empyema, and died 3 months after initiation of chemoimmunotherapy.

Patients with symptomatic brain metastases and/or use of corticosteroids at the time of treatment initiation did not have a worse outcome and even trended toward a better outcome with a median OS of 17.1 months compared with 12.6 months in the overall population (Supplementary Table 1). Further analyses are summarized in Supplementary Table 1.

As previously described, we have included patients into group A who had minor deviations from the eligibility criteria of KN-189 on the basis of predetermined criteria. There were 12 patients included in group A based on the following tolerance criteria: 10 patients finished brain radiotherapy within less than 7 days before treatment initiation; in three patients, corticosteroids were discontinued within 10 days after initiation of treatment; and one patient with anemia at treatment initiation received a transfusion in the first 10 days after initiation of therapy. In this subgroup of patients, median PFS was 5.2 months, median OS was 9.8 months, and ORR was 42%. This unplanned subgroup analysis was not statistically analyzed for significant differences. Nevertheless, it seems that the outcome in these patients with minor deviations is worse than that of the other patients in group A and more similar to that of patients in group B, suggesting that even minor deviations from the inclusion criteria of the trial negatively affect the outcome of patients receiving combined chemoimmunotherapy.

### Modified Analysis

All patients have received a minimum of one dose of platinum-pemetrexed doublet chemotherapy. Six patients have died before the start of pembrolizumab therapy. Five patients have received the KN-189 regimen as an individual treatment approach after previous lines of palliative systemic treatment for advanced lung cancer (three patients had previous single-agent pembrolizumab and two patients had previous dabrafenib/trametinib). We have performed a modified survival analysis excluding these pretreated patients and patients who have not received at least one dose of pembrolizumab. Results did not differ from the overall analysis. Median PFS was 9.8 months (95% CI: 5.8–13.7) for patients in group A in the modified analysis as compared with 5.3 months (95% CI: 1.8–8.8) for patients in group B (*p* = 0.16) (Supplementary Fig. 1). Median OS was 16.5 months (95% CI: 7.5–25.6) for patients in group A in the modified analysis as compared with 7.4 months (95% CI: 0.0–18.5) for patients in group B (*p* = 0.16) (Supplementary Fig. 2). As in the overall analysis, ORR differed significantly between groups A and B (63% versus 35%, *p* = 0.001).

## Discussion

More than half of all patients (61%) treated according to the KN-189 regimen at our center would not have met the inclusion criteria of KN-189 as defined by the trial protocol. This percentage is surprisingly high. It reveals that the real-world population of patients with metastatic NSCLC significantly and relevantly differs from patient populations treated in clinical trials who are intended to define the treatment standard. Patients who did not meet the eligibility criteria tended to have worse outcomes. Subgroup analysis indicated that this detrimental effect seemed to be driven by patients with poor performance status and/or active infection, who accounted for 43% of patients in group B.

Median PFS and OS for treatment with chemotherapy in combination with pembrolizumab reported by KN-189 were 9 and 22 months, respectively.^8^ In comparison, median PFS in our total population was 7 months and median OS was 12.6 months. The more adequate comparison is between KN-189 and group A of our study population, as they have identical eligibility criteria. PFS and OS was 9.2 months and 16.5 months, respectively, in group A of our study. ORR reported by KN-189 was 48.3% compared with an ORR of 43% in our total population and 59% in group A. Median PFS, OS, and ORR in group A of this real-world study are thus overall comparable with KN-189.

Real-world evidence for combined immunochemotherapy is scarce, and most analyses include only small patient numbers. Comparing these studies is difficult owing to different selection criteria and patient populations. Real-world PFS has been reported in the range of 6.4 to 11.6 months and real-world OS in the range of 12.9 to 23.1 months.^13^, ^14^, ^15^, ^16^ In summary, reported outcomes for the KN-189 treatment regimen from real-world data tend to be inferior to the reported trial results. To our knowledge, no previous study has taken the approach of this analysis considering all patients having received combined platinum-based chemoimmunotherapy according to KN-189. The unique novelty of this analysis is the fact that we included all patients treated at a university hospital according to KN-189 and analyzed the results depending on the adherence to the inclusion criteria for KN-189. Therefore, this analysis offers the unique opportunity to assess the benefit of this treatment regimen in a real-world patient population and in subgroups that are frequent and relevant in daily clinical routine.

As in our study, ECOG PS greater than or equal to 2 has previously been identified as an independent adverse risk factor in patients receiving combined immunochemotherapy.^14^ Parikh et al.^17^ have analyzed survival outcomes following immune checkpoint inhibitor (ICI) therapy among trial-ineligible patients with different solid cancers in which trial ineligibility was defined as ECOG PS greater than or equal to 2 or the presence of kidney or liver dysfunction. They could not find any survival benefit of monoimmunotherapy or combined immunochemotherapy compared with non-ICI regimens in their overall population; however, a specific subgroup analysis for patients with NSCLC was not reported. A preliminary safety analysis of the SAKK 19/17 trial investigating first-line durvalumab in patients with metastatic NSCLC, programmed death-ligand 1 expression greater than or equal to 25%, and ECOG PS of 2 has reported an unexpectedly high number of early deaths from rapid tumor progression during the early accrual phase.^18^ The trial was amended to exclude patients with highly symptomatic tumor burden and has since finished accrual. In contrast, recently reported results from the IPSOS study have revealed that patients with lung cancer with ECOG PS greater than or equal to 2 may have a benefit from first-line immunotherapy.^9^ The role of immunotherapy and combined immunochemotherapy in patients with a poor performance status remains thus to be defined.

In addition to poor performance status, we have found active infections at the time of treatment initiation to be significantly associated with an adverse outcome. Although these risk factors are known, it can sometimes be difficult in daily practice to differentiate between tumor-related and constitutional symptoms, and individual treatment decisions are made by patients and physicians. The same applies for other patients who are underrepresented in clinical trials but should not in general be precluded from treatment, for example, patients with prior disease or substance abuse. Nevertheless, our data suggest that these treatment decisions must be made carefully. Clinical practice at our center includes a mandatory discussion of all patients with a de novo diagnosis of lung cancer at the regular multidisciplinary board for thoracic tumors. Patients are generally evaluated by two physicians at the time of diagnosis and for the planning of systemic treatment, including a senior specialist.

This study has limitations. First, this was a retrospective analysis with a small patient population and thus low statistical power. Larger retrospective data sets are needed to confirm the trends identified in this study. Second, owing to our approach to include *all* patients treated with the KN-189 regimen at our hospital, we have included pretreated patients, thus precluding comparability with KN-189 and other retrospective studies to some extent. We have accounted for this by reporting a modified analysis that excluded pretreated patients. Third, dividing patients retrospectively according to hypothetical trial eligibility will never fully represent the conditions of prospective randomization. It must be assumed that trial conditions lead to a positive selection of patients even beyond eligibility criteria, because fitter and more compliant patients are considered and referred for clinical trials a priori.

In conclusions, our study provides a comprehensive retrospective single-center analysis of all patients treated with platinum, pemetrexed, and pembrolizumab, most of which would have been trial ineligible. Our data reveal that the results of KN-189 can be reproduced to some extent in real-world clinical practice if the same criteria of patient selection are applied as in the clinical trial. Nevertheless, the realities of daily practice differ substantially from trial conditions and many treatment choices are made off label. We have undertaken an unprecedented retrospective head-to-head comparison of trial-eligible and ineligible patients treated with the KN-189 regimen, expectably finding a worse outcome in the latter group as a trend. Significant risk factors in this group are a poor performance status and/or active infection at the time of treatment initiation. The results of this study indicate that we must be very careful when expanding indications to trial-ineligible patients, because we could be harming them rather than helping. We need more and larger retrospective studies to get a better idea which patients truly benefit. More importantly, we urgently need prospective evidence on whether and how to treat vulnerable patient groups as frequently seen in daily practice.

## CRediT Authorship Contribution Statement

**Till Wallrabenstein, Sacha I. Rothschild:** Conception and study design.

**Till Wallrabenstein, David König, Maximilian Mamot, Sacha I. Rothschild:** Development of methodology.

**Maximilian Mamot, Till Wallrabenstein, Eveline Daetwyler:** Data acquisition.

**Till Wallrabenstein, Maximilian Mamot, Eveline Daetwyler, David König, Sacha I. Rothschild:** Data analysis and interpretation.

**Till Wallrabenstein:** Statistical analysis.

**Till Wallrabenstein, Maximilian Mamot:** Writing—original draft.

**Till Wallrabenstein, Maximilian Mamot, Eveline Daetwyler, David König, Sacha I. Rothschild:** Writing—review and editing.

**Sacha I. Rothschild:** Study supervision.

## References

[1] Siegel R.L., Miller K.D., Jemal A. (2020). Cancer statistics, 2020. CA Cancer J Clin.

[2] Chen V.W., Ruiz B.A., Hsieh M.C., Wu X.C., Ries L.A.G., Lewis D.R. (2014). Analysis of stage and clinical/prognostic factors for lung cancer from SEER registries: AJCC staging and collaborative stage data collection system. Cancer.

[3] NSCLC Meta-Analyses Collaborative Group (2008). Chemotherapy in addition to supportive care improves survival in advanced non–small-cell lung cancer: a systematic review and meta-analysis of individual patient data from 16 randomized controlled trials. J Clin Oncol.

[4] Schiller J.H., Harrington D., Belani C.P. (2002). Comparison of four chemotherapy regimens for advanced non–small-cell lung cancer. N Engl J Med.

[5] Hanna N.H., Robinson A.G., Temin S. (2021). Therapy for Stage IV non–small-cell lung cancer with driver alterations: ASCO and OH (CCO) joint guideline update. J Clin Oncol.

[6] Hanna N.H., Schneider B.J., Temin S. (2020). Therapy for Stage IV non–small-cell lung cancer without driver alterations: ASCO and OH (CCO) joint guideline update. J Clin Oncol.

[7] Gandhi L., Rodríguez-Abreu D., Gadgeel S. (2018). Pembrolizumab plus chemotherapy in metastatic non–small-cell lung cancer. N Engl J Med.

[8] Rodríguez-Abreu D., Powell S.F., Hochmair M.J. (2021). Pemetrexed plus platinum with or without pembrolizumab in patients with previously untreated metastatic nonsquamous NSCLC: protocol-specified final analysis from KEYNOTE-189. Ann Oncol.

[9] Lee S.M., Schulz C., Prabhash K. (2022). LBA11 - IPSOS: results from a phase III study of first-line (1L) atezolizumab (atezo) vs single-agent chemotherapy (chemo) in patients (pts) with NSCLC not eligible for a platinum-containing regimen. Ann Oncol.

[10] Felip E., Ardizzoni A., Ciuleanu T. (2020). CheckMate 171: A phase 2 trial of nivolumab in patients with previously treated advanced squamous non-small cell lung cancer, including ECOG PS 2 and elderly populations. Eur J Cancer.

[11] Altan M., Singhi E.K., Worst M. (2022). Clinical effectiveness and safety of anti-PD-(L)1 therapy among older adults with advanced non-small cell lung cancer. Clin Lung Cancer.

[12] Eisenhauer E.A., Therasse P., Bogaerts J. (2009). New response evaluation criteria in solid tumours: Revised RECIST guideline (version 1.1). Eur J Cancer.

[13] Velcheti V., Hu X., Piperdi B., Burke T. (2021). Real-world outcomes of first-line pembrolizumab plus pemetrexed-carboplatin for metastatic nonsquamous NSCLC at US oncology practices. Sci Rep.

[14] Morimoto K., Yamada T., Yokoi T. (2021). Clinical impact of pembrolizumab combined with chemotherapy in elderly patients with advanced non-small-cell lung cancer. Lung Cancer.

[15] Zhang J., Wu D., Zhang Z. (2021). Pembrolizumab or bevacizumab plus chemotherapy as first-line treatment of advanced nonsquamous nonsmall cell lung cancer: a retrospective cohort study. Technol Cancer Res Treat.

[16] Kehl K.L., Greenwald S., Chamoun N.G., Manberg P.J., Schrag D. (2021). Association between first-line immune checkpoint inhibition and survival for Medicare-insured patients with advanced non–small cell lung cancer. JAMA Netw Open.

[17] Parikh R.B., Min E.J., Wileyto E.P. (2021). Uptake and survival outcomes following immune checkpoint inhibitor therapy among trial-ineligible patients with advanced solid cancers. JAMA Oncol.

[18] Mark M., Froesch P., Eboulet E.I. (2021). SAKK 19/17: safety analysis of first-line durvalumab in patients with PD-L1 positive, advanced nonsmall cell lung cancer and a performance status of 2. Cancer Immunol Immunother.

