# Supplementary material for: Real-World Data of Combined Immunochemotherapy in Patients With Nonsquamous Advanced NSCLC. A Single-Center Retrospective Study
Source: JTO Clin Res Rep. 2023 Mar 24;4(5):100509. doi: 10.1016/j.jtocrr.2023.100509 (PMC10164892; doi:10.1016/j.jtocrr.2023.100509)
Supplement: Supplementary Materials [file mmc1.docx]

**SUPPLEMENTARY MATERIAL**

*Supplementary Figure A*


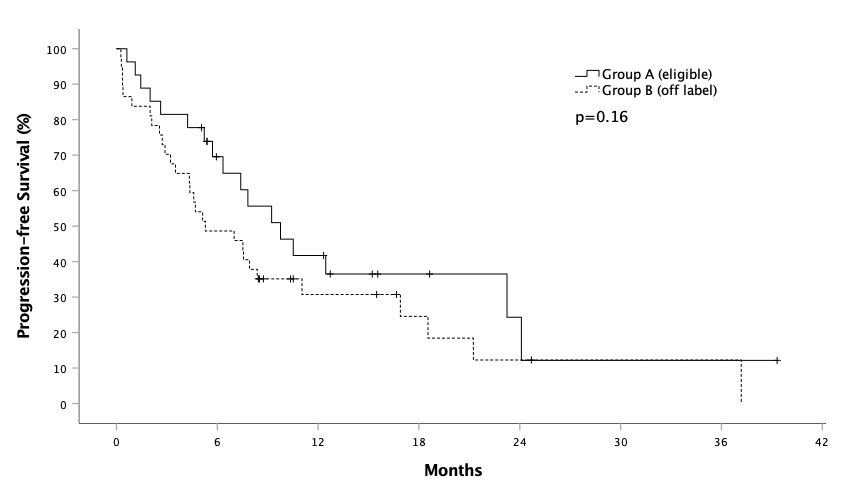


Progression-free survival of 64 patients with metastatic non-small cell lung cancer treated with platinum, pemetrexed and pembrolizumab. Patients in group A would retrospectively have fulfilled selection criteria of KEYNOTE-189. Patients in group B would have been trial-ineligible. This modified analysis excludes patients who have not received a at least one dose of each drug (triple therapy) and patients having received previous lines of systemic treatment. p=0.16.

*Supplementary Figure B*
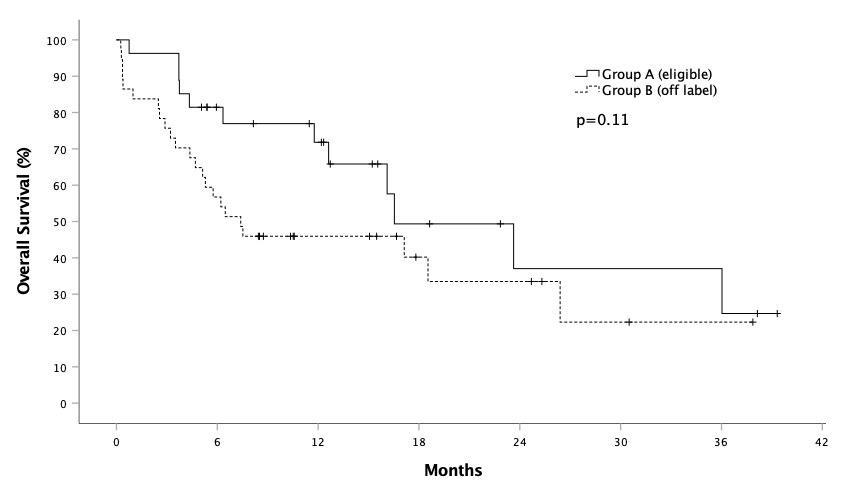


Overall survival of 64 patients with metastatic non-small cell lung cancer treated with platinum, pemetrexed and pembrolizumab. Patients in group A would retrospectively have fulfilled selection criteria of KEYNOTE-189. Patients in group B would have been trial-ineligible. This modified analysis excludes patients who have not received a at least one dose of each drug (triple therapy) and patients having received previous lines of systemic treatment. p=0.11.

*Supplementary Table A. Secondary endpoints including subgroup analysis*

| Characteristic | Group A (n=29) | Group B (n=46) | TOTAL (n=75) | ECOG≥2  (n=18) | On steroids (n=10) | Unstable symptomatic brain metastases  (n=5) | p |
| --- | --- | --- | --- | --- | --- | --- | --- |
| Average number of platinum based chemotherapy cycles | 3.4 | 3 | 3.2 | 2.8 | 3.4 | 3.8 | 0.13 |
| Completed 4 cycles of platinum based chemotherapy *(%)* | 20 *(69.0)* | 24 *(52.2)* | 44 *(58.7)* | 8 *(44.4)* | 7 *(70.0)* | 4 *(80.0)* | 0.23 |
| Cisplatin-based *(%)* | 1 *(3.4)* | 5 *(10.9)* | 6 *(8.0)* | 1 *(5.6)* | 1 *(10.0)* | 1 *(20.0)* | 0.40 |
| Carboplatin-based *(%)* | 28 *(96.6)* | 41 *(89.1)* | 69 *(92.0)* | 17 *(94.4)* | 9 *(90.0)* | 4 *(80.0)* | 0.40 |
| Patients receiving maintenance pemetrexed *(%)* | 18 *(62.1)* | 19 *(41.3)* | 37 *(49.3)* | 6 *(33.3)* | 6 *(60.0)* | 3 *(60.0)* | 0.10 |
| Average number of cycles maintenance pemetrexed | 4.3 | 2.8 | 3.4 | 1.7 | 5.9 | 3.8 | 0.22 |
| Median/*Average* number of cycles IO | 7 / *8.8* | 4 / *7.3* | 5 / *7.9* | 3.5 / *5.6* | 7 / *8.7* | 7 / *7.2* | 0.16 |
| Outcome measures |  |  |  |  |  |  |  |
| Median follow up for primary endpoint (PFS) according to reverse Kaplan Meyer | 15.6 (95% CI 11.5-19.6) | 15.5 (95% CI 10.8-20.1) | 15.6 (95% CI 12.4-18.7) | 10.4 (95% CI 5.2-15.5) | 15.5 (95% CI 9.3-21.7) | 10.4 (NA) | 0.73 |
| Median follow up for secondary endpoint (OS) according to reverse Kaplan Meyer | 15.6 (95% CI 10.0-21.2) | 16.7 (95% CI 13.0-20.3) | 16.7 (95% CI 13.2-20.1) | 13.8 (95% CI 2.7-24.8) | 16.7 (95% CI 7.8-25.5) | 24.7 (95% CI 1.8-47.6) | 0.81 |
| PFS, RECIST1.1 (months) | 9.2 (95% CI 5.7-12.8) | 4.6 (95% CI 2.6-6.6) | 7 (95% CI 4.6-9.4) | 4.3 (95% CI 1.3-7.4) | 7 (95% CI 2.0-12.0) | 7.6 (95% CI 6.4-8.8) | 0.12 |
| OS, RECIST1.1 (months) | 16.5 (95% CI 6.9-26.2) | 6.5 (95% CI 4.0-9.0) | 12.6 (95% CI 4.2-21.1) | 5.1 (95% CI 0.0-11.0) | 17.1 (95% CI 0.0-35.6) | NR | 0.11 |
| EFS, RECIST1.1 (months) | 5.3 (95% CI 3.2-7.3) | 2.7 (95% CI 1.8-3.6) | 4.1(95%CI 2.4-5.8) | 2.5 (95% CI 1.0-4.0 | 4.3 (95% CI 0.1-8.6) | 5.5 (95% CI 0.0-11.3) | 0.34 |
| Time on treatment (months) | 5.7 (95%CI 4.4-7.0) | 3.1 (95% CI 0.6-5.6) | 4.7 (95%CI 3.2-6.3) | 2.5 (95% CI 0.9-4.0) | 5.5 (95% CI 5.5-10.8) | 5.5 (95% CI 0.0-11.3) | 0.3 |
| OS from time of diagnosis of stage IV disease, RECIST1.1 (months) | 25.7 (95% CI 9.5-42.0) | 8.4 (95% CI 0.0-17.4) | 16.9 (95% CI 6.5-27.3) | 8.1 (95% CI 4.6-11.5) | 21.8 (95% CI 0.0-47.2) | NR | 0.19 |
| Overall response rate *(%)* | 17 *(58.6)* | 15 *(32.6)* | 32 *(42.7)* | 4 *(22.2)* | 4 *(40.0)* | 3 *(60.0)* | 0.03 |
| Complete remission *(%)* | 0 *(0.0)* | 4 *(8.7)* | 4 *(5.3)* | 1 *(5.6)* | 0 *(0.0)* | 0 *(0.0)* | 0.15 |
| Partial remission *(%)* | 17 (58.6) | 11 *(23.9)* | 28 *(37.3)* | 3 *(16.7)* | 4 *(40.0)* | 3 *(60.0)* | 0.003 |
| Stable disease *(%)* | 5 *(17.2)* | 15 *(32.6)* | 20 *(26.7)* | 7 *(38.9)* | 3 *(30.0)* | 1 *(20.0)* | 0.18 |
| Progressive disease *(%)* | 6 *(20.7)* | 8 *(17.4)* | 14 *(18.7)* | 3 *(16.7)* | 1 *(10.0)* | 0 *(0.0)* | 0.77 |
| Early death *(%)* | 1 *(3.4)* | 8 *(17.4)* | 9 *(12.0)* | 4 *(22.2)* | 2 *(20.0)* | 1 *(20.0)* | 0.14 |
| Median/*average* duration of response | 14.1 (95%CI 0.0-29.9), *16.7 (95%CI 8.2-25.1)* | 8.2 (95%CI 0.0-20.2), *15.8 (95%CI 8.1-23.5)* | 14.1 (95%CI 4.1-24.0), *16.5 (95%CI 11.0-22.1)* | NR, *6.8 (95% CI 6.1-7.5)* | NR, *18.6 (95% CI 11.5-25.8)* | NR, *17.2 (95% CI 8.3-26.2)* | 0.66 |
| Toxicity |  |  |  |  |  |  |  |
| Immune related adverse events *(%)* | 8 *(27.6)* | 6 *(13.0)* | 14 *(18.7)* | 1 *(5.6)* | 2 *(20.0)* | 2 *(40.0)* | 0.14 |
| Grade 3 or higher irAE *(%)* | 2 *(6.9)* | 1 *(2.2)* | 3 *(4.0)* | 0 *(0.0)* | 0 *(0.0)* | 0 | 0.56 |
| Discontinuation of IO due to irAE *(%)* | 5 *(17.2)* | 4 *(8.7)* | 9 *(12.0)* | 0 *(0.0)* | 2 *(20.0)* | 2 *(40.0)* | 0.30 |
| Discontinuation of IO due to patient wish *(%)* | 1 *(3.4)* | 2 *(4.3)* | 3 *(4.0)* | 1 *(5.6)* | 0 *(0.0)* | 0 *(0.0)* | 1 |
| Discontinuation of platinum based chemotherapy due to any toxicity *(%)* | 1 *(3.4)* | 1 *(2.2)* | 2 *(2.7)* | 0 *(0.0)* | 0 *(0.0)* | 0 *(0.0)* | 1 |
| Discontinuation of platinum based chemotherapy due to patient wish *(%)* | 2 *(6.9)* | 2 *(4.3)* | 4 *(5.3)* | 1 *(5.6)* | 1 *(10.0)* | 0 *(0.0)* | 0.64 |
| Discontinuation of maintenance chemotherapy due to any toxicity *(%)* | 5 *(17.2)* | 8 *(17.4)* | 13 *(17.3)* | 0 *(0.0)* | 1 *(10.0)* | 1 *(20.0)* | 1 |
| Further treatment lines after Carbo/Cis-Pem-Pem |  |  |  |  |  |  |  |
| 1 *(%)* | 6 *(20.7)* | 4 *(8.7)* | 10 *(13.3)* | 0 *(0.0)* | 1 *(10.0)* | 0 *(0.0)* | 0.171 |
| 2 or more *(%)* | 1 *(3.4)* | 4 *(8.7)* | 5 *(6.7)* | 3 *(16.7)* | 2 *(20.0)* | 1 *(20.0)* | 0.643 |

Treatment characteristics and outcome measures of 75 patients with metastatic non-small cell lung cancer treated with platinum, pemetrexed and pembrolizumab. Patients in group A would retrospectively have fulfilled selection criteria of KEYNOTE-189. Patients in group B would have been trial-ineligible. Subgroup analysis are descriptive and have not been statistically explored. Given p values refer to inter-group comparison of group A and B only).

*Supplementary Table B. Multivariate analysis*

| Variabel | HR | 95% CI | P |
| --- | --- | --- | --- |
| Age | 0.993 | 0.950-1.038 | 0.76 |
| Sex | 1.307 | 0.643-2.656 | 0.46 |
| Histology (adenocarcinoma vs. other) | 1.100 | 0.369-3.275 | 0.87 |
| Low performance status (ECOG ≥ 2) | 2.250 | 1.038-4.874 | 0.04 |
| Unstabe symptomatic brain metastasis | 0.679 | 0.097-4.759 | 0.70 |
| Active infection | 20.703 | 2.548-168.194 | 0.005 |
| Relevant malignant co-disease | 0.822 | 0.311-2.168 | 0.69 |
| Cardiovascular co-disease | 0.642 | 0.319-1.295 | 0.22 |
| Pulmonary co-disease | 1.250 | 0.569-2.746 | 0.58 |
| Autoimmune disease | 2.008 | 0.604-6.671 | 0.26 |
| Previous systemic therapy | 0.970 | 0.253-3.715 | 0.96 |
| Previous curative surgery | 0.816 | 0.360-1.850 | 0.63 |
| Previous surgery for stage IV disease | 1.170 | 0.566-2.415 | 0.67 |
| Previous curative radio-chemotherapy | 2.571 | 0.302-21.916 | 0.39 |
| Previous radiotherapy for stage IV disease | 0.852 | 0.401-1.808 | 0.68 |
| Steroid dependency/co-medication | 0.502 | 0.120-2.099 | 0.35 |
| Use of antibiotics up to 4 weeks before treatment initiation | 0.392 | 0.072-2.136 | 0.28 |
| Use of antibiotics during treatment | 0.803 | 0.404-1.595 | 0.53 |

Multivariate analysis by Cox regression regarding prognostic impact of patient-related, disease-related and treatment-related variables on primary endpoint progression free survival (PFS). Low performance status and concurrent active infection during treatment initiation were significantly associated with shorter PFS.

*Supplementary Table C. Multivariate analysis*

| Variabel | HR | 95% CI | p |
| --- | --- | --- | --- |
| Age | 1.005 | 0.960-1.053 | 0.83 |
| Sex | 1.612 | 0.701-3.711 | 0.26 |
| Histology (adenocarcinoma vs. other) | 0.547 | 0.144-2.088 | 0.38 |
| Low performance status (ECOG ≥ 2) | 2.333 | 0.986-5.524 | 0.054 |
| Unstabe symptomatic brain metastasis | 0.375 | 0.038-3.728 | 0.40 |
| Active infection | 20.471 | 2.119-197.769 | 0.01 |
| Relevant malignant co-disease | 0.835 | 0.289-2.411 | 0.74 |
| Cardiovascular co-disease | 0.682 | 0.315-1.478 | 0.33 |
| Pulmonary co-disease | 1.342 | 0.589-3.055 | 0.48 |
| Autoimmune disease | 0.621 | 0.126-3.065 | 0.56 |
| Previous systemic therapy | 0.758 | 0.167-3.445 | 0.72 |
| Previous curative surgery | 0.599 | 0.240-1.496 | 0.27 |
| Previous surgery for stage IV disease | 1.033 | 0.476-2.241 | 0.94 |
| Previous curative radio-chemotherapy | 1.407 | 0.156-12.675 | 0.76 |
| Previous radiotherapy for stage IV disease | 0.789 | 0.327-1.903 | 0.60 |
| Steroid dependency/co-medication | 0.666 | 0.147-3.026 | 0.60 |
| Use of antibiotics up to 4 weeks before treatment initiation | 0.736 | 0.121-4.474 | 0.74 |
| Use of antibiotics during treatment | 0.866 | 0.395-1.902 | 0.72 |

Multivariate analysis by Cox regression regarding prognostic impact of patient-related, disease-related and treatment-related variables on secondary endpoint overall survival (OS). As a trend low performance status was a negative prognostic factor. Concurrent active infection during treatment initiation was significantly associated with shorter OS.

*Supplementary Table D: Multivariate analysis*

| Variabel | Odds ratio | 95% CI | p |
| --- | --- | --- | --- |
| Age | 0.987 | 0.913-1.068 | 0.75 |
| Sex | 2.507 | 0.617-10.192 | 0.20 |
| Histology (adenocarcinoma vs. other) | 1.252 | 0.153-10.261 | 0.83 |
| Low performance status (ECOG ≥ 2) | 0.260 | 0.055-1.231 | 0.09 |
| Unstabe symptomatic brain metastasis | 7.917 | 0.138-454.126 | 0.32 |
| Active infection | 0.0 | NC | 0.999 |
| Relevant malignant co-disease | 0.870 | 0.160-4.737 | 0.87 |
| Cardiovascular co-disease | 0.484 | 0.142-1.652 | 0.25 |
| Pulmonary co-disease | 0.701 | 0.181-2.713 | 0.61 |
| Autoimmune disease | 3.421 | 0.246-47.586 | 0.36 |
| Previous systemic therapy | 0.216 | 0.012-3.904 | 0.30 |
| Previous curative surgery | 2.615 | 0.542-12.623 | 0.23 |
| Previous surgery for stage IV disease | 0.518 | 0.138-1.946 | 0.33 |
| Previous curative radio-chemotherapy | 0.0 | - | 0.999 |
| Previous radiotherapy for stage IV disease | 1.428 | 0.315-6.470 | 0.64 |
| Steroid dependency/co-medication | 0.343 | 0.014-8.551 | 0.51 |
| Use of antibiotics up to 4 weeks before treatment initiation | 1.595 | 0.081-31.552 | 0.76 |
| Use of antibiotics during treatment | 2.083 | 0.541-8.021 | 0.29 |

Multivariate analysis by logistic regression regarding prognostic impact of patient-related, disease-related and treatment-related variables on secondary endpoint overall response rate (ORR). Low performance status and concurrent active infection during treatment initiation were significantly associated with shorter PFS. An odds ratio <1 indicates a lower likelihood of response.
